# Supplementary material for: Predation and fragmentation portrayed in the statistical structure of prey time series
Source: BMC Ecol. 2009 May 6;9:10. doi: 10.1186/1472-6785-9-10 (PMC2689204; doi:10.1186/1472-6785-9-10)
Supplement: Additional file 2 — Voles and related classes ODDox Documentation. ODDox documentation of the agent-based model (ALMaSS) applied by Hendrichsen et al. The documentation is started by activating main.html. [file 1472-6785-9-10-S2.zip › Vole_ODDox/struct_farm_event-members.html]

ALMaSS ODDox: Member List

- Main Page
- Related Pages
- Classes
- Files

- Alphabetical List
- Class List
- Class Hierarchy
- Class Members

# FarmEvent Member List

This is the complete list of members for FarmEvent, including all inherited members.

|  |  |  |
| --- | --- | --- |
| FarmEvent(TTypesOfVegetation a\_event, LE \*a\_field, int a\_todo, long a\_run, bool a\_lock, int a\_start, bool a\_first\_year, TTypesOfVegetation a\_nextcrop) | FarmEvent | `[inline]` |
| m\_event | FarmEvent |  |
| m\_field | FarmEvent |  |
| m\_first\_year | FarmEvent |  |
| m\_lock | FarmEvent |  |
| m\_next\_tov | FarmEvent |  |
| m\_run | FarmEvent |  |
| m\_startday | FarmEvent |  |
| m\_todo | FarmEvent |  |

---

Generated on Thu Jan 22 14:13:46 2009 for ALMaSS ODDox by 
 1.5.6
